# 二代测序技术检测儿童急性淋巴细胞白血病的基因突变谱及其预后意义

**DOI:** 10.3760/cma.j.issn.0253-2727.2022.01.005

**Published:** 2022-01

**Authors:** 湧智 郑, 浩 郑, 再生 陈, 雪玲 华, 少华 乐, 健 李, 建达 胡

**Affiliations:** 1 福建医科大学附属协和医院小儿血液科，福州 350001 Department of Pediatric Hematology, Fujian Institute of Hematology, Fujian Provincial Key Laboratory, Fujian Medical University Union Hospital, Fuzhou 350001, China; 2 福建医科大学附属协和医院血液科，福建省血液病研究所，福建省血液病学重点实验室，福州 350001 Department of Hematology, Fujian Institute of Hematology, Fujian Provincial Key Laboratory, Fujian Medical University Union Hospital, Fuzhou 350001, China

**Keywords:** 白血病，淋巴细胞，急性, 基因突变, 二代测序, 儿童, Leukemia,lymphoblastic,acute, Genetic muation, Next-generation sequencing, Child

## Abstract

**目的:**

分析儿童急性淋巴细胞白血病（ALL）的基因突变谱及其预后意义。

**方法:**

回顾性分析2016年11月至2019年12月福建医科大学附属协和医院采用二代测序技术（NGS）行基因突变检测的141例初治ALL患儿的临床资料，分析基因突变谱及其对ALL患儿预后的影响。

**结果:**

141例患儿中，83例（58.9％）检出体细胞突变，包括37个Ⅰ类及123个Ⅱ类突变位点。单核苷酸变异（SNV）为最常见的突变类型。KRAS（20/160，12.5％）为最常见的突变基因，其次为NOTCH1（11.9％）及NRAS（10.6％）。RAS通路（KRAS、FLT3、PTPN11）、PAX5及TP53突变仅在B-ALL患儿中检出，而FBXW7、PTEN突变仅在T-ALL患儿中检出；NRAS突变主要在B-ALL中检出，而NOTCH1突变主要在T-ALL中检出。每例T-ALL患儿检出的平均基因突变个数显著高于B-ALL患儿（4.16±1.33对2.04±0.92，*P*＝0.004）。按照有无遗传变异将患儿分为突变组和无突变组，两组的性别、年龄、初诊白细胞计数、微小残留病监测结果、预计3年无事件生存（EFS）率及总生存（OS）率差异均无统计学意义（*P*值均>0.05）；但突变组T-ALL以及融合基因阴性患儿的比例显著高于无突变组（*P*值分别为0.021和<0.001）。进一步亚组分析，在融合基因阴性的患儿中，有Ⅰ类突变的患儿预计3年EFS率显著低于无Ⅰ类突变的患儿（85.5％对100.0％，*P*＝0.039）；在B-ALL患儿中，伴TP53突变的患儿预计3年EFS率显著低于不伴有TP53突变的患儿（37.5％对91.2％，*P*<0.001）。

**结论:**

体细胞突变在儿童ALL中较为常见，与临床表型及预后具有一定的相关性，NGS可作为传统MICM分型检查的重要补充。

## Abstract (en)

**Objective:**

This study analyzed the correlation between genetic mutation and prognostic significance in childhood acute lymphoblastic leukemia (ALL).

**Methods:**

Targeted exome by next-generation sequencing (NGS) technology was used to carry out molecular profiling of untreated 141 children with ALL in Fujian Medical University Union Hospital from November 2016 to December 2019. Correlation of genetic features and clinical features and outcomes was analyzed.

**Results:**

Among the 141 pediatric patients with ALL, 160 somatic mutations were detected in 83 patients (58.9％), including 37 grade Ⅰ mutations and 123 grade Ⅱ mutations. Single nucleotide variation was the most common type of mutation. KRAS was the most common mutant gene (12.5％), followed by NOTCH1 (11.9％), and NRAS (10.6％). RAS pathway (KRAS, FLT3, PTPN11), PAX5 and TP53 mutations were only detected, and NRAS mutations was mainly found in B-ALL while FBXW7 and PTEN mutations were only found, and NOTCH1 mutation was mainly detected in T-ALL. The average number of mutations detected in each child with T-ALL was significantly higher than in children with B-ALL (4.16±1.33 *vs* 2.04±0.92, *P*＝0.004). The children were divided into mutation and non-mutation groups according to the presence or absence of genetic variation. There were no statistically significant differences in sex, age, newly diagnosed white blood cell count, minimal or measurable residual disease monitoring results, expected 3-year event-free survival (EFS) and overall survival (OS) between the two groups (*P*>0.05). On the other hand, the proportion of T-ALL and fusion gene negative children in the mutant group was significantly higher than the non-mutation group (*P*＝0.021 and 0.000, respectively). Among the patients without fusion gene, the EFS of children with grade I mutation was significantly lower than children without grade I mutation (85.5％ *vs* 100.0％, *P*＝0.039). Among children with B-ALL, the EFS of those with TP53 mutation was significantly lower than those without TP53 mutation (37.5％ *vs* 91.2％, *P*<0.001).

**Conclusion:**

Genetic variation is more common in childhood ALL and has a certain correlation with clinical phenotype and prognosis. Therefore, targeted exome by NGS can be used as an important supplement to the traditional morphology, immunology, cytogenetics, and molecular biology classification．

急性淋巴细胞白血病（ALL）是儿童最常见的恶性肿瘤，约占同时期儿童恶性肿瘤的三分之一[1]。目前在发达国家，儿童ALL的5年无事件生存（EFS）率可达85％以上，总生存（OS）率可达90％以上[1]–[3]；在我国，儿童ALL的5年EFS率也达到了80％，OS率达到了85％以上[4]。儿童ALL的治愈率不断提高很大程度上归功于根据初诊危险因素和最小残留病（MRD）评估确定的早期治疗反应对患者进行更精确的危险度分层治疗；另外与预后和治疗相关遗传学异常的不断发现并对其进行有效的靶向治疗也起到了重要的作用[2]。

在儿童ALL中，一些融合基因和染色体异常在疾病发生发展过程中起到了关键作用，而且与预后相关性强，已被作为危险度分层的依据[5]–[6]。近年来研究发现体细胞基因突变也与儿童ALL的疾病表型、临床特征及预后具有相关性，了解基因突变与临床特征、早期疗效和远期预后等的相关性，有助于我们探讨其临床意义和发现新的治疗靶点[7]–[10]。随着二代测序（next-generation sequencing, NGS）技术的发展，进行基因全外显子（whole-exome sequencing, WES）检测可同时完成多个癌症相关基因测序，这是比单基因检测更具时间和成本效益的基因检测策略[11]。本研究采用NGS对初诊ALL患儿进行全外显子基因检测，探讨体细胞基因突变与疾病表型、临床特征及预后的相关性。

## 病例与方法

一、一般资料

2016年11月至2019年12月在福建医科大学附属协和医院小儿血液科初诊并进行血液肿瘤全外显子基因检测的141例ALL患儿入组本研究，其中男89例，女52例，中位年龄5.0（0.8～13.6）岁，B-ALL 126例（89.4％），T-ALL 15例（10.6％）。所有入组患儿在化疗前除了进行骨髓细胞形态学、免疫分型、染色体核型、融合基因（MICM）分型检测，另外还抽取3 ml骨髓（EDTA抗凝管）送检血液肿瘤全外显子基因检测。

二、血液肿瘤全外显子基因检测方法

1. 测序方法：采用Covaris超声波高性能样品处理系统将质量合格的基因组DNA随机打断成150～250 bp左右的片段，使用美国安捷伦（Agilent）公司+6Agilent Sureselect Human All Exon V6试剂盒进行外显子富集，通过Illumina HiSeq平台对合格的文库进行高通量测序，保证每个样品的数据量达标。测序得到的原始图像数据经Illumina HiSeq碱基识别软件（Base Calling）转化为原始序列数据（raw reads），即双末端reads（paired-end reads），数据以FASTQ文件格式存储，称之为raw data。

2. 分析流程：①首先对原始下机数据（raw data）进行过滤和质控，得到满足质控要求的clean data。②对clean data使用Burrows-WheelerAligne（r BWA）软件，与人参考基因组（GRCh37/HG19）进行比对。比对结果使用Picard-tools去除PCR重复片段。基于比对结果，对每个样品的测序深度、覆盖度、比对率等评价指标进行统计。③使用Genome Analysis Toolkit（GATK）软件进行SNP和小片段插入/缺失检测，使用Annovar软件对变异结果做注释。

3. 变异筛选流程：①关注外显子区和剪接位点的变异，主要包括功能缺失型变异（获得终止密码子的突变、移码突变和关键剪接位点突变）、错义突变、非移码的缺失/插入；②过滤掉可能因为基因组序列重复导致的假阳性位点，如位于串联重复序列或基因组上存在高度相似性序列的重复序列区域的位点；③去掉正常人群中常见的多态性位点，如在1000 genome、EXAC等数据库中满足东亚人群等位基因突变频率大于1％的突变位点。这类位点被认为是患者本身正常细胞也携带的多态性位点，而不是肿瘤特有的体细胞突变。

4. 结果解读：经过筛选过滤后的基因突变位点的临床分级解读依照2017年AMP/ASCO/ACMG/CAP协作发表的肿瘤序列变异解读指南[12]；筛选出临床等级为Ⅰ类（已被FDA批准的治疗或者已收录在专业指南中或该领域专家具有一致意见的强有力研究）、Ⅱ类（在其他肿瘤中已被FDA批准的治疗或者研究中的疗法；或者多个已经发表的小型研究具有一些共识，或临床前试验或小型、无共识的案例报道）的突变。

5. 突变验证：①筛选的变异具有Ⅰ级和Ⅱ级临床意义；②利用IGV可视化工具，查看候选的变异是否真实；③从BAM文件中随机选取相关比对reads进行blast核实变异；④采用sanger测序等方法进行验证。

6. 体细胞突变与胚系突变的鉴别：对于无法区分所有检测到的突变点位为体细胞或生殖系的改变，特别是突变丰度接近50％或100％的变异位点的患者，取口腔黏膜细胞及缓解期骨髓作为正常对照的样本以进行鉴别。

三、诊断标准及治疗方案

2例诊断后放弃治疗，1例诊断后转院治疗，1例早期死亡，其余137例接受规范治疗，27例参照儿童ALL诊疗建议（第四次修订）[6]进行ALL诊断及临床危险度分层治疗，110例参照中国儿童癌症协作组（Chinese Children's Cancer Group, CCCG）ALL 2015方案[13]进行ALL诊断及临床危险度分层治疗。根据初诊危险因素及MRD检测结果，分为低危、中危及高危组[14]。血液肿瘤全外显子基因检测结果不作为制定危险度分层治疗方案的依据。

四、随访

采用查阅病历或电话的方式进行随访。随访截至2020年8月15日。137例（突变组80例，无突变组57例）规范治疗的患儿纳入生存分析。总生存（OS）期为患者开始治疗至死亡或末次随访的时间；无事件生存（EFS）期为开始治疗至发生任何事件的时间，包括任何原因所致死亡、疾病进展、复发或失访。纳入生存分析的患儿中无失访病例。

五、统计学处理

采用回顾性分析方法，用SPSS 21.0软件进行统计分析，非正态分布样本集中趋势用中位数表示，计量样本比较用秩和检验，两个样本率的比较用卡方检验或Fisher确切概率法，采用Kaplan-Meier方法分析突变和无突变组的OS和EFS，并行Log-rank检验。*P*<0.05为差异有统计学意义。

## 结果

1. 儿童ALL体细胞突变检出情况：141例患儿中，83例（58.9％）检出体细胞突变，包括37个Ⅰ类突变（涉及基因包括IKZF1、TP53、CREBBP、KRAS、JAK1）及123个Ⅱ类突变。共检出36种基因突变，12种突变（包括CTCF、EP300、FAT1、JAK1、JAK3、KMT2A、NR3C1、NT5C2、PDGFRB、STAT5B、TET2、WT1）仅在1例患儿中出现，其余24种突变至少在2例患儿中出现（图1A）。突变类型以单核苷酸变异（SNV）最为常见，占79.4％，其次为无义突变（stopgain）、移码插入/缺失等。127个SNV的突变基因中，核苷酸变异类型以C>T最为常见，占28.3％（36/127），其次为G>A、A>G等。160个基因突变位点中，KRAS（20/160，12.5％）为最常见的突变，其次为NOTCH1（11.9％）、NRAS（10.6％）、CREBBP（5.6％）、FLT3（5.6％）、PTPN11（5.6％），17种最常见的基因突变位点及突变类型如图1B所示。KRAS、FLT3、PTPN11、PAX5及TP53突变仅在B-ALL患儿中检出（检出率分别为24.1％、9.6％、9.6％、3.6％、6.0％），而FBXW7、PTEN突变仅在T-ALL患儿中检出（检出率分别为33.3％、13.3％）；NRAS突变主要在B-ALL中检出（B-ALL中检出率为15.7％；T-ALL中检出率为6.7％），而NOTCH1突变主要在T-ALL中检出（T-ALL中检出率80％，B-ALL检出率为4.8％），突变基因与免疫表型的相关性如图1C所示。83例检出突变的患儿中，13例T-ALL患儿检出45个突变位点，70例B-ALL患儿检出115个突变位点，每例T-ALL患儿检出的平均突变个数显著高于B-ALL患儿（4.16±1.33对2.04±0.92，*P*＝0.004）。

**图1:**
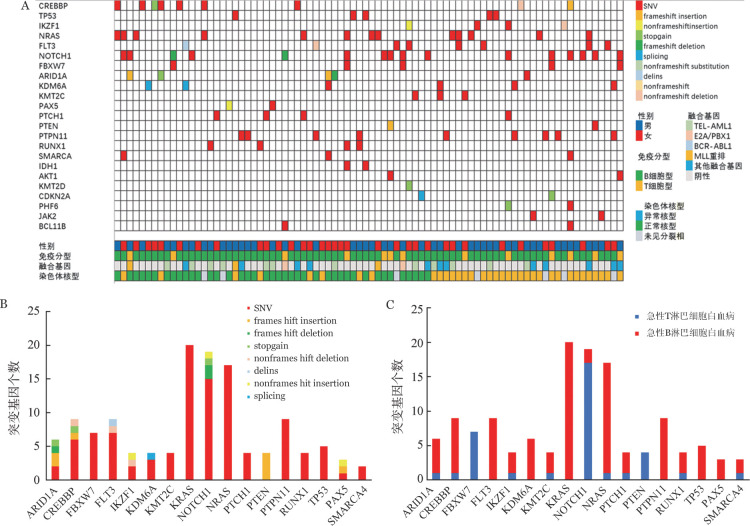
83例检出体细胞突变的急性淋巴细胞白血病患儿的基因突变情况 A：基因瀑布图（仅显示至少在2例以上患者中出现的突变基因），在上部表格中，每一行表示一个基因，每个颜色表示一种突变类型；B：17种最常见的基因突变位点及突变类型；C：不同免疫表型的常见突变基因。SNV：单核苷酸变异

2. 体细胞突变与儿童ALL临床特征的相关性：按照有无突变将患儿分为突变组（83例）和无突变组（58例），两组临床特征的比较如表1所示。两组的性别、中位年龄、≥10岁患者比例、初诊中位WBC、WBC≥50×10^9^/L患者比例，以及伴异常核型的比例差异均无统计学意义（*P*值均>0.05）。但突变组融合基因阴性患儿的比例显著高于无突变组（*P*<0.001）。融合基因阴性患儿突变发生率为72.9％（54/74），而融合基因阳性患儿突变发生率为43.3％（29/67），前者显著高于后者（*P*<0.001）。对融合基因阳性的患儿进一步行亚组分析，结果显示：突变组TEL-AML1、E2A-PBX1、BCR-ABL融合基因阳性的比例显著低于无突变组（*P*值分别为0.018、0.027、0.004）。

**表1:** 儿童急性淋巴细胞白血病（ALL）中体细胞突变与临床特征的相关性

临床特征	总体（141例）	突变组（83例）	无突变组（58例）	*χ*^2^值	*P*值
年龄［岁，*M*（范围）］	5（0.8~13.6）	5（0.8~13.6）	5（1.6~13.5）		0.984^a^
≥10岁患者［例（％）］	21（14.9）	12（14.5）	9（15.5）	0.030	0.862
性别［例（％）］				2.425	0.119
男	89（63.1）	48（57.8）	41（70.7）		
女	52（36.9）	35（42.2）	17（29.3）		
诊断时WBC［×10^9^/L，*M*（范围）］	9.71（0.88~765.18）	8.31（1.39~765.18）	11.02（0.88~495.90）		0.809^a^
WBC≥50×10^9^/L［例（％）］	37（26.2）	24（28.9）	13（22.4）	0.746	0.388
融合基因				12.800	0.000
阴性	74（52.5）	54（65.1）	20（34.5）		
阳性	67（47.5）	29（34.9）	38（65.5）		
TEL-AML1	30（21.3）	12（14.5）	18（31.0）	5.601	0.018
E2A/PBX1	4（2.8）	0（0）	4（6.9）		0.027^b^
BCR-ABL1	6（4.3）	0（0）	6（10.3）		0.004^b^
MLL重排	5（3.5）	4（4.8）	1（1.7）		0.328^b^
SIL-TAL1	3（2.1）	3（3.6）	0（0）		0.268^b^
其他融合基因	19（13.5）	10（12.0）	9（15.5）	0.352	0.553
染色体核型				0.422	0.810
正常	99（70.2）	60（72.3）	39（67.2）		
异常	29（20.6）	16（19.3）	13（22.4）		
无法获得	13（9.2）	7（8.4）	6（10.3）		

注：^a^因不符合正态分布，应用非参数检验的独立样本中位数检验；^b^应用Fisher精确检验

3. 体细胞突变与儿童ALL早期疗效及预后的相关性：突变组和无突变组在诱导化疗中MRD<1％的比例（突变组75.0％，无突变组78.9％，*P*＝0.590），以及诱导化疗后MRD<0.01％的比例（突变组87.5％，无突变组84.2％，*P*＝0.583）差异均无统计学意义。中位随访15.3（6.6～45.8）个月，8例（5.8％）复发，复发中位时间为8.2（3.8～18.8）个月。复发后，2例放弃治疗，6例再治疗：1例再诱导后重症感染死亡，5例再诱导后行造血干细胞移植，1例移植后再复发死亡，4例移植后仍在随访。137例患儿预计3年EFS率及OS率分别为89.8％和96.5％（图2A）。突变组和无突变组的预计3年EFS率（90.3％对90.1％，*P*＝0.805）及OS率（96.8％对96.1％，*P*＝0.705）差异均无统计学意义（图2B、C）。进一步进行亚组分析，有Ⅰ类突变的患儿预计3年EFS率低于无Ⅰ类突变的患儿，但差异无统计学意义（84.6％对91.2％，*P*＝0.271）（图2D）；在融合基因阴性患儿中，有Ⅰ类突变的患儿预计3年EFS率显著低于无Ⅰ类突变的患儿（85.5％％对100.0％，*P*＝0.039）（图2E）。B-ALL患儿中，伴TP53突变的患儿预计3年EFS率显著低于不伴有TP53突变的患儿（37.5％对91.2％，*P*<0.001）（图2F），但有无KRAS、NARS、CREBBP突变对EFS均无明显影响。

**图2:**
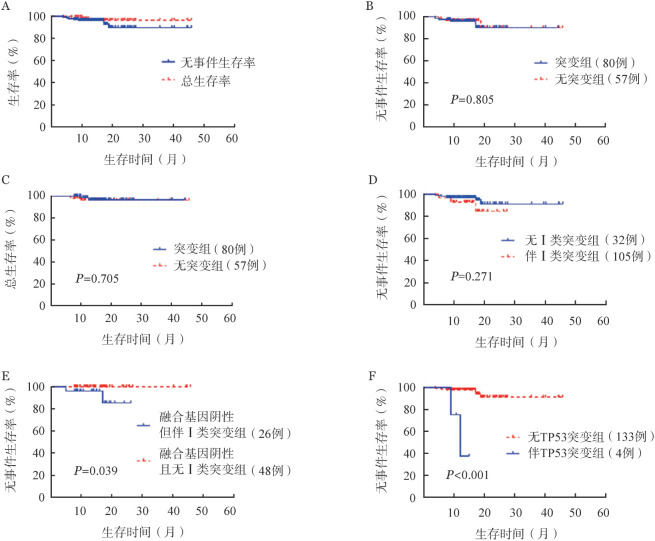
基因突变对儿童急性淋巴细胞白血病（ALL）无事件生存率和总生存率的影响 A：137例规范治疗ALL患儿的生存曲线；B：伴或不伴基因突变组无事件生存曲线；C：伴或不伴基因突变组的总生存曲线；D：伴或不伴Ⅰ类基因突变组的无事件生存曲线；E：融合基因阴性伴或不伴基Ⅰ类因突变组的无事件生存曲线；F：B-ALL患儿中伴或不伴TP53基因突变组无事件生存曲线

## 讨论

儿童ALL是一类具有高度异质性的疾病，其诊疗需要结合MICM分型进行危险度分层，其中分子生物学异常主要包括融合基因、基因突变及基因异常表达[15]。NGS作为检测基因突变的分子生物学技术，具有通量高、灵敏度高、成本低等优势，在成人及儿童白血病的诊断分型、预后判断、指导治疗、MRD监测及克隆演变等方面具有重要的价值[2],[16]–[18]。

较多研究结果显示，RAS通路（KRAS、NRAS、PTPN11、FLT3）、CREBBP突变在B-ALL复发时检出率高，而且与不良预后相关[19]–[21]。基于以上研究，将以上突变作为不良预后的标志。在未治疗B-ALL患儿中，RAS通路基因突变也是最常见的突变，但不影响预后[7],[21]。在本研究中，所有测序对象均为未治疗的ALL患儿，KRAS、NRAS、PTPN11、FLT3、CREBBP突变为B-ALL最常见的突变基因，对预后无显著影响，与上述研究结果一致[7],[22]。但本研究中，融合基因阴性的B-ALL突变发生率高于融合基因阳性组，而且对融合基因阴性B-ALL进行亚组预后分析显示，有Ⅰ类突变（包括KRAS、CREBBP、TP53）的患儿EFS率显著低于无Ⅰ类突变的患儿，表明在融合基因阴性亚型中，以上基因突变可能预后不良，这也为融合基因阴性患儿的预后判断提供了新的方法和思路。

TP53突变与人类超过50％癌症的发生发展密切相关[23]。在一项纳入625例成人ALL的研究中，TP53突变的总体发生率为15.7％，在低二倍体、MYC重排亚型中更为常见，且与不良预后相关[9]。在一项纳入3801例儿童B-ALL的研究中，TP53突变的检出率为2％，TP53突变组年龄显著高于无TP53突变组，且预后更差[24]。Zhang等[7]报道，TP53突变在未治疗B-ALL中的发生率为3.5％（4/114），TP53突变型ALL患儿预计3年无复发生存率为（33.3±27.2）％，显著低于TP53野生型ALL患儿。在本研究中，TP53突变在B-ALL中的发生率显著低于TP53野生型ALL，与以上报道相符。

在T-ALL患儿中，NOTCH1、FBXW7突变发生率分别为>50％及8％～30％[8],[24]–[27]。在德国BFM95/2000及我国BCH-2003、CCLG-2008研究中，伴NOTCH1/FBXW7突变的儿童T-ALL长期生存率更高[25],[27]–[28]，但荷兰DCOG及其与德国合作的COALL-97研究中，伴NOTCH1/FBXW7突变的T-ALL患儿对泼尼松反应更好，但长期生存率却没有更高[26]。另外，我国学者发现，NOTCH1突变T-ALL患者预后还受到CDKN2A/B缺失的影响，两者同时存在OS也较差[29]。在本研究中，NOTCH1、FBXW7突变在T-ALL的发生率分别为80％及33.3％，略高于报道，可能与样本量较小有关；伴NOTCH1/FBXW7突变的T-ALL患儿EFS率较高，与报道相符，但由于T-ALL病例数过少，免疫分型比例的偏差可能会影响突变谱，以及预后分析的统计学效能。肿瘤抑制基因PTEN在儿童和成人T-ALL的发生率分别为18％和14％，对预后的影响仍有争议，主要与PTEN失活机制有关[18],[30]。Jenkinson等[31]报道，PTEN突变不影响T-ALL的预后。Tesio等[30]报道，大片段缺失致PTEN突变的儿童T-ALL预后差，而微缺失致PTEN突变对预后则无影响。本研究中，PTEN突变在T-ALL的发生率为13.3％，低于文献报道，考虑与样本量小有关。另外，在本研究中，T-ALL突变频率高于B-ALL，这与Zhang等[7],[22]的报道结果相一致。

总之，遗传变异在儿童ALL中较为常见，B-ALL与T-ALL的突变谱系及频率有明显的差别；尤其在融合基因阴性的ALL患儿中，突变可能与预后相关。因此，NGS靶向检测全外显子可作为ALL儿童MICM分型的重要补充，但是否可作为初诊危险度分层的依据尚需进一步研究。
